# Supplementary material for: Significant Changes in Serum MicroRNAs after High Tibial Osteotomy in Medial Compartmental Knee Osteoarthritis: Potential Prognostic Biomarkers
Source: Diagnostics (Basel). 2021 Feb 7;11(2):258. doi: 10.3390/diagnostics11020258 (PMC7914593; doi:10.3390/diagnostics11020258)
Supplement: Supplementary file 1 [file diagnostics-11-00258-s001.pdf]

Table S1. The expression levels of all miRNAs in the serum at profiling stage.

miRNA	Pre-op.		Post-op. month 6		Fold regulation	p-value
	ΔCt	$2^{-\Delta Ct}$	ΔCt	$2^{-\Delta Ct}$		
**hsa-miR-19b-3p**	−0.66 ± 0.87	1.57	−2.47 ± 1.38	5.53	**3.51**	**0.011**
**hsa-miR-29c-3p**	−0.32 ± 0.79	1.25	−2.10 ± 1.26	4.28	**3.43**	**0.015**
**hsa-miR-424-5p**	−0.48 ± 1.17	1.39	−2.23 ± 0.80	4.69	**3.37**	**0.021**
hsa-miR-16-5p	−5.79 ± 1.08	55.20	−7.31 ± 1.20	159.01	2.88	0.074
hsa-miR-29a-3p	−1.42 ± 0.40	2.68	−2.10 ± 0.97	4.28	1.6	0.081
hsa-miR-23a-3p	−5.28 ± 2.77	38.76	−7.40 ± 1.34	168.39	4.34	0.091
hsa-miR-195-5p	−5.20 ± 1.38	36.82	−6.80 ± 1.79	111.45	3.03	0.091
hsa-miR-30a-5p	−3.21 ± 1.12	9.28	−4.82 ± 1.75	28.21	3.04	0.11
hsa-miR-145-5p	0.54 ± 0.58	0.69	−0.35 ± 1.19	1.28	1.85	0.11
hsa-miR-125a-5p	−3.16 ± 1.29	8.96	−4.65 ± 1.78	25.09	2.8	0.113
hsa-miR-181b-5p	−0.49 ± 1.13	1.40	−1.58 ± 0.68	2.98	2.12	0.115
hsa-miR-93-5p	−2.16 ± 1.19	4.45	−3.62 ± 1.56	12.28	2.76	0.14
hsa-miR-30d-5p	−2.94 ± 1.18	7.65	−4.59 ± 1.18	24.03	3.14	0.165
hsa-miR-17-5p	−1.42 ± 1.29	2.67	−2.44 ± 1.38	5.43	2.03	0.167
hsa-miR-543	0.51 ± 0.29	0.7	0.88 ± 0.46	0.54	−1.3	0.171
hsa-let-7i-5p	−1.73 ± 1.37	3.31	−2.87 ± 1.76	7.33	2.21	0.184
hsa-miR-130a-3p	−0.50 ± 1.00	1.41	−1.46 ± 0.93	2.75	1.95	0.193
hsa-miR-20a-5p	−2.91 ± 0.89	7.52	−3.72 ± 1.82	13.14	1.75	0.194
hsa-miR-106b-5p	−0.80 ± 1.18	1.75	−1.85 ± 2.12	3.61	2.07	0.2
hsa-miR-15b-5p	−3.31 ± 1.74	9.91	−4.46 ± 2.12	22.00	2.22	0.203
hsa-miR-19a-3p	−1.51 ± 1.02	2.85	−2.40 ± 1.48	5.27	1.85	0.205
hsa-miR-125b-5p	−1.57 ±1.51	2.98	−2.79 ± 1.61	6.90	2.32	0.21
hsa-miR-20b-5p	−1.64 ± 1.18	3.13	−2.57 ± 1.54	5.92	1.89	0.214
hsa-miR-29b-3p	−0.01 ± 1.11	1.01	−1.04 ± 1.20	2.06	2.04	0.221
hsa-miR-520d-3p	0.67 ± 0.82	0.63	1.06 ± 0.49	0.48	−1.31	0.238
hsa-miR-98-5p	0.52 ± 0.55	0.7	0.00 ± 0.94	1	1.44	0.238
hsa-miR-30c-5p	−2.13 ± 1.26	4.37	−3.06 ± 1.61	8.36	1.91	0.239
hsa-miR-101-3p	0.61 ± 0.45	0.65	0.14 ± 1.30	0.91	1.39	0.245

hsa-miR-23b-3p	−2.79 ± 1.50	6.94	−3.75 ± 1.56	13.42	1.94	0.25
hsa-miR-374a-5p	−0.34 ± 0.67	1.27	−1.07 ± 1.04	2.11	1.66	0.25
hsa-miR-30e-5p	−2.70 ± 1.14	6.51	−3.48 ± 1.99	11.14	1.71	0.252
hsa-let-7c-5p	−2.78 ± 1.06	6.88	−3.39 ± 0.91	10.46	1.52	0.285
hsa-miR-449a	0.66 ± 0.65	0.63	0.97 ± 0.42	0.51	−1.24	0.305
hsa-let-7d-5p	−3.44 ± 0.90	10.84	−3.97 ± 1.48	15.67	1.45	0.324
hsa-let-7g-5p	−2.29 ± 1.23	4.89	−2.75 ± 1.79	6.71	1.37	0.326
hsa-miR-34a-5p	0.76 ± 0.63	0.59	1.06 ± 0.49	0.48	−1.23	0.334
hsa-miR-186-5p	−1.00 ± 0.91	2.00	−1.29 ± 1.76	2.45	1.23	0.345
hsa-miR-128-3p	−0.35 ± 0.79	1.28	−0.81 ± 1.52	1.75	1.37	0.365
hsa-miR-1324	0.79 ± 0.70	0.58	1.06 ± 0.49	0.48	−1.2	0.368
hsa-miR-300	0.79 ± 0.70	0.58	1.06 ± 0.49	0.48	−1.2	0.368
hsa-miR-302a-3p	0.79 ± 0.70	0.58	1.06 ± 0.49	0.48	−1.2	0.368
hsa-miR-302b-3p	0.79 ± 0.70	0.58	1.06 ± 0.49	0.48	−1.2	0.368
hsa-miR-302c-3p	0.79 ± 0.70	0.58	1.06 ± 0.49	0.48	−1.2	0.368
hsa-miR-34c-5p	0.79 ± 0.70	0.58	1.06 ± 0.49	0.48	−1.2	0.368
hsa-miR-372-3p	0.79 ± 0.70	0.58	1.06 ± 0.49	0.48	−1.2	0.368
hsa-miR-381-3p	0.79 ± 0.70	0.58	1.06 ± 0.49	0.48	−1.2	0.368
hsa-miR-410-3p	0.79 ± 0.70	0.58	1.06 ± 0.49	0.48	−1.2	0.368
hsa-miR-449b-5p	0.79 ± 0.70	0.58	1.06 ± 0.49	0.48	−1.2	0.368
hsa-miR-497-5p	0.79 ± 0.70	0.58	1.06 ± 0.49	0.48	−1.2	0.368
hsa-miR-513b-5p	0.79 ± 0.70	0.58	1.06 ± 0.49	0.48	−1.2	0.368
hsa-miR-519c-3p	0.79 ± 0.70	0.58	1.06 ± 0.49	0.48	−1.2	0.368
hsa-miR-519d-3p	0.79 ± 0.70	0.58	1.06 ± 0.49	0.48	−1.2	0.368
hsa-miR-524-5p	0.79 ± 0.70	0.58	1.06 ± 0.49	0.48	−1.2	0.368
hsa-miR-545-3p	0.79 ± 0.70	0.58	1.06 ± 0.49	0.48	−1.2	0.368
hsa-miR-548c-3p	0.79 ± 0.70	0.58	1.06 ± 0.49	0.48	−1.2	0.368
hsa-miR-548d-3p	0.79 ± 0.70	0.58	1.06 ± 0.49	0.48	−1.2	0.368
hsa-miR-548e-3p	0.79 ± 0.70	0.58	1.06 ± 0.49	0.48	−1.2	0.368
hsa-miR-590-5p	0.79 ± 0.70	0.58	1.06 ± 0.49	0.48	−1.2	0.368
hsa-miR-607	0.79 ± 0.70	0.58	1.06 ± 0.49	0.48	−1.2	0.368

hsa-miR-655-3p	0.79 ± 0.70	0.58	1.06 ± 0.49	0.48	−1.2	0.368
hsa-miR-875-3p	0.79 ± 0.70	0.58	1.06 ± 0.49	0.48	−1.2	0.368
hsa-miR-9-5p	0.79 ± 0.70	0.58	1.06 ± 0.49	0.48	−1.2	0.368
hsa-miR-340-5p	0.79 ± 0.70	0.58	1.05 ± 0.49	0.48	−1.2	0.378
hsa-miR-21-5p	−5.78 ± 1.68	54.81	−6.81 ± 1.42	111.94	2.04	0.404
hsa-let-7e-5p	−1.59 ± 0.98	3.02	−1.91 ± 1.44	3.77	1.25	0.439
hsa-miR-301b-3p	0.79 ± 0.70	0.58	0.97 ± 0.42	0.51	−1.13	0.462
hsa-miR-181a-5p	0.50 ± 0.44	0.71	0.32 ± 1.08	0.8	1.13	0.468
hsa-let-7a-5p	−3.24 ± 1.80	9.44	−3.90 ± 1.75	14.96	1.59	0.491
hsa-miR-301a-3p	0.79 ± 0.70	0.58	0.97 ± 0.49	0.51	−1.13	0.504
hsa-miR-373-3p	0.79 ± 0.70	0.58	0.96 ± 0.49	0.51	−1.12	0.515
hsa-miR-511-5p	0.79 ± 0.70	0.58	0.95 ± 0.50	0.52	−1.12	0.536
hsa-miR-15a-5p	0.74 ± 0.71	0.6	0.64 ± 1.04	0.64	1.07	0.586
hsa-miR-656-3p	−1.37 ± 0.57	2.59	−1.11 ± 0.69	2.16	−1.2	0.599
hsa-let-7f-5p	−3.29 ± 1.41	9.76	−3.55 ± 1.64	11.75	1.2	0.607
hsa-miR-30b-5p	−0.92 ± 1.26	1.89	−1.26 ± 1.39	2.40	1.27	0.63
hsa-miR-144-3p	−0.79 ± 1.67	1.73	−0.63 ± 1.49	1.54	−1.12	0.658
hsa-let-7b-5p	−6.39 ± 1.62	84.00	−7.25 ± 0.66	152.54	1.82	0.664
hsa-miR-202-3p	0.52 ± 0.80	0.7	0.46 ± 0.93	0.73	1.05	0.735
hsa-miR-181d-5p	0.73 ± 0.57	0.6	0.65 ± 0.53	0.64	1.06	0.792
hsa-miR-211-5p	0.79 ± 0.70	0.58	0.66 ± 0.50	0.63	1.09	0.798
hsa-miR-130b-3p	−0.42 ± 1.77	1.34	−0.37 ± 1.44	1.30	−1.03	0.811
hsa-miR-520e	0.79 ± 0.70	0.58	0.79 ± 0.84	0.58	1	0.82
hsa-miR-454-3p	−0.35 ± 1.02	1.28	−0.59 ± 0.76	1.51	1.18	0.872
hsa-miR-181c-5p	0.79 ± 0.70	0.58	0.74 ± 0.45	0.6	1.04	0.983

Values are presented as the mean ± standard deviation. *$p < 0.05$

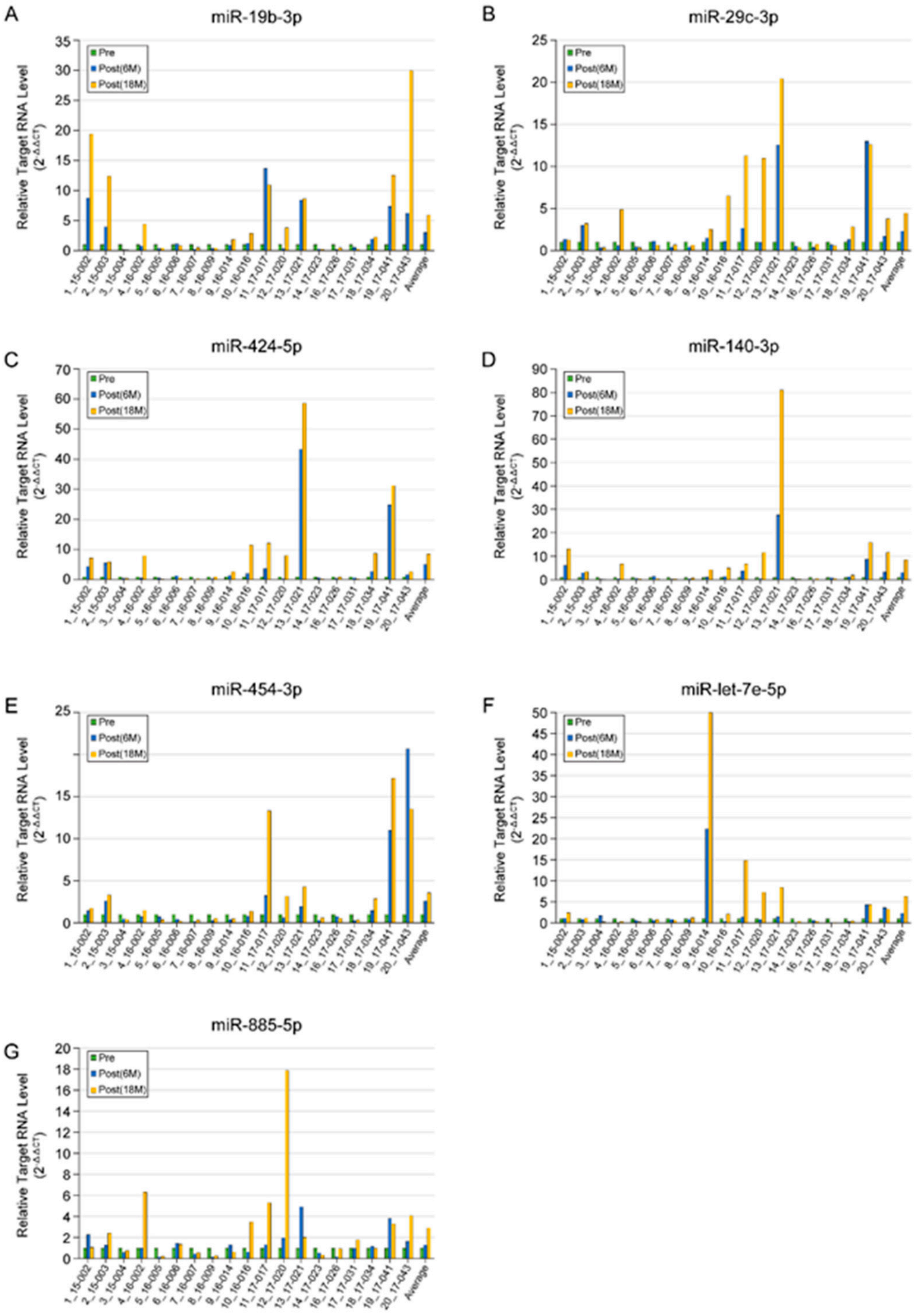

Figure S1. Raw data of miR-19b-3p (A), miR-29c-3p (B), miR-424-5p (C), miR-140-3p (D), miR-454-3p (E), miR-let-7e-5p (F), and miR-885-5p (G) from all patients prior to HTO and 6 and 18 months after HTO.
